# Influence of Different Legume Seeds on the Development, Oviposition, and Digestive Enzyme Activity of *Callosobruchus chinensis* L. (Coleoptera: Chrysomelidae)

**DOI:** 10.3390/insects17060556

**Published:** 2026-05-28

**Authors:** Tao Zhang, Keying Wang, Maria K. Sakka, Rongrong Yuan, Lingyan Jian, Xinshuo Hu, Chun Wang, Christos G. Athanassiou, Yu Cao

**Affiliations:** 1 School of Pharmacy, Guizhou University of Traditional Chinese Medicine, Guiyang 550025, China; 2 Guizhou Provincial Key Laboratory for Rare Animal and Economic Insect of the Mountainous Region, Guizhou Key Laboratory of Agricultural Biosecurity, Guiyang University, Guiyang 550005, China; 3 Laboratory of Entomology and Agricultural Zoology, Department of Agriculture, Crop Production and Rural Environment, School of Agricultural Sciences, University of Thessaly, 384 46 Nea Ionia, Greece; msakka@uth.gr

**Keywords:** *Callosobruchus chinensis*, population development, digestive enzyme, bean seeds, host adaptability, Leguminosae

## Abstract

In this study, the effects of five bean seeds (*Vigna radiata*, *Pisum sativum*, *Vigna unguiculata*, *Vicia faba*, and *Glycine max*) on the development, survival, oviposition, and digestive enzyme activities of *C. chinensis* were investigated. The duration of *C. chinensis* development from egg to adult was shortest when reared on *V. radiata* (26.97 d) and longest on *G. max* (30.92 d) (*p* < 0.01). Similarly, the emergence rate of *C. chinensis* was highest on *V. radiata* (66.11%) and lowest on *G. max* (33.89%) (*p* < 0.01). Significant differences in the fecundity of *C. chinensis* were also detected, with the highest values on *V. radiata* (63.36 eggs/female), followed by *P. sativum* (54.27 eggs/female), *V. unguiculata* (51.30 eggs/female), *V. faba* (42.47 eggs/female), and *G. max* (37.29 eggs/female) (*p* < 0.01). For an equal initial insect number, after continuous rearing for 30, 60, or 90 d, there were significantly more offspring on *V. radiata* than on the other species, with the fewest offspring detected on *G. max* (*p* < 0.01). Although CL activity (*p* = 0.33) in *C. chinensis* did not differ significantly among the different legume species, activities of PEP (*p* < 0.01) and α-AMS (*p* < 0.01) in *C. chinensis* were all highest on *V. radiata* and lowest on *G. max,* and activities of LPS (*p* < 0.01) were highest on *G. max* and lowest on *V. radiata*. The differences in population dynamics associated with different bean seeds may be related to the activity of particular digestive enzymes, which play important roles in insect nutrient metabolism and growth. Our results indicated that *V. radiata* is the most suitable host diet, whereas *G. max* is the least suitable for the development of *C. chinensis*.

## Simple Summary

The cowpea beetle, *Callosobruchus chinensis* (L.) (Coleoptera: Chrysomelidae), is a major pest of legume seeds. Different food sources will affect the physiological and ecological characteristics of insects. Here, we investigated the population development and digestive enzyme activities of *C. chinensis* reared on five different legume seeds: *Vigna radiata*, *Pisum sativum*, *Vigna unguiculata*, *Vicia faba*, and *Glycine max*. The results showed that *C. chinensis* had the shortest developmental period, highest adult survival rate, and highest fecundity when fed on *V. radiata.* For an equal initial number of adults, in the continuous rearing treatments (30, 60, or 90 d, respectively) on these bean seeds, *C. chinensis* showed a significantly larger population on *V. radiata*, while the smallest on *G. max*. In addition, activities of pepsin (PEP) and *α*-amylase (*α*-AMS) in *C. chinensis* were the highest when feeding on *V. radiata*, but the lowest on *G. max*. The activity of lipase (LPS) in *C. chinensis* was the highest when feeding on *G. max*, while there were no significant differences in the cellulase (CL) activities when fed on different bean seeds. Our results indicate that different food sources significantly influenced the metabolism of *C. chinensis*, which in turn may affect the population performance of insects. These findings will not only facilitate the accurate prediction of *C. chinensis* occurrences on different stored bean seeds, but also deepen our understanding of the host adaptation of *C. chinensis* and related adaptive mechanisms in physiological ecology.

## 1. Introduction

The cowpea beetle, *Callosobruchus chinensis* (L.) (Coleoptera: Bruchidae), a widespread and oligophagous pest of legumes (Leguminosae), causes quantitative and qualitative losses and degradations in seeds [1,2]. It is widely distributed throughout the tropical and subtropical regions of the world [3,4]. The species is an important post-harvest pest of stored grains, such as cowpea, lentil, pea, green gram, and other legumes [5,6]. Damage to stored bean seeds leads to weight loss, low viability of seeds, and low nutritional quality [4,7].

*Callosobruchus chinensis* lays eggs on the surface of stored grains. The larvae and pupae of *C. chinensis* live inside of a single grain and cause damage by feeding [6,8]. Stored grains can be lost in a few months due to the relatively rapid development, high reproductive capacity, and continual generations of *C. chinensis* [4,8]. Unless measures are taken early enough, the infestation by the larvae of this species can be devastating, particularly due to the fact that, as internal feeders, larvae are not much affected by measures that are applied at the external part of the kernel.

Chemical fumigants and insecticides are often used to protect stored grains against *C. chinensis* during storage; however, these compounds may leave residues on stored grains that eventually will affect the environment, animals and humans [9,10]. Chemical insecticides could lead to the development of resistance in these stored products’ pests, making control more difficult [10,11]. Nevertheless, one of the solutions suggested is the utilization of legume species and genotypes that are affected less by the infestation, a method that is generally known as “varietal resistance”, and has been utilized in the case of several grain species, particularly cereals [12].

Legumes are vital plant-based sources of nutrients, particularly protein, and rank as the second most important food source for humans following cereals. In China, a variety of legume cultivars are widely cultivated and consumed, including *Vigna radiata*, *Pisum sativum*, *Vigna unguiculata*, *Vicia faba*, and *Glycine max* (all belonging to the order Fabales and family Fabaceae). In this study, the development, survival, fecundity, and population growth of this pest on these five different stored bean seeds were investigated to explore the host preference and adaptation of *C. chinensis*. In addition, digestive enzymes were measured to examine the physiological adaptation of *C. chinensis* to the different legume species. These results improve our understanding of the variation in damage caused by *C. chinensis* on different stored bean seeds, illustrating its food preferences and providing insights into host adaptation from the perspective of physiological ecology. Furthermore, these data for the population dynamics of *C. chinensis* on different bean cultivars provide a basis for pest control or improved IPM programs.

## 2. Materials and Methods

### 2.1. Insect Rearing

*Callosobruchus chinensis* was reared in glass jars (5 L) covered with muslin cloth to prevent insect escape and ensure ventilation, as described in our previous studies [13,14]. This species has been cultured in our laboratory (Guizhou Provincial Key Laboratory for Rare Animal and Economic Insect of the Mountainous Region, Guiyang University, Guizhou, China) since 2022 and is maintained on *Vigna angularis* (Fabales: Fabaceae), with a moisture content of 12–14%. This insect colony was kept in a climate chamber, at 25 ± 1 °C, 65 ± 5% RH, and under a 14: 10 h (light/dark) photoperiod.

### 2.2. Stored Bean Seeds

Five stored leguminous products, *V. radiata*, *P. sativum*, *V. unguiculata*, *V. faba*, and *G. max* (with moisture contents of 10–13%, 12–14%, 10–13%, 10–13%, and 12–14%, respectively), were purchased from the Guiyang Grain Commodity Market (Guiyang City, China) and were kept without pesticides.

### 2.3. Development of Immature Stages and Emergence Rate of C. chinensis

Groups of 200 *C. chinensis* (males and females) were placed into glass jars (2.5 L) for oviposition. Each jar contained 20.0 g of one of the five stored bean seeds. After 24 h, *C. chinensis* adults were removed, and newly laid eggs on the stored materials were carefully transferred into separate Petri dishes (60 mm in diameter, 30 mm in height). Sixty eggs from each stored product culture were checked daily until the emergence of larvae [13,15]. Fresh stored material for rearing was provided. As the larvae and pupae of *C. chinensis* feed inside of beans, developmental periods were defined as the time until eclosion (i.e., the emergence of *C. chinensis* adults). The emergence rate of *C. chinensis* was calculated as the number of emerged adults relative to the initial egg count. There were three replicates for these observations on each stored bean. These investigations were conducted at 25 ± 1 °C, 65 ± 5% RH, and under a 14:10 h (light/dark) photoperiod in a climate chamber.

### 2.4. Oviposition

Newly emerged *C. chinensis* adults from each bean seed in the above experiment were used to assess fecundity, according to our previous study [13]. All adults were paired (1 female: 1 male) and were transferred into Petri dishes (6 cm in diameter, 3 cm in height), with one pair per Petri dish, containing one of the five uninfested bean seeds (10.0 g) for oviposition. These paired *C. chinensis* adults were transferred daily to new Petri dishes containing fresh food materials, and all materials were checked daily to assess egg laying. Fecundity (i.e., the number of eggs laid during the reproductive period) was recorded for each female *C. chinensis* until death. The offspring were reared to adulthood for sex determination and to calculate the female sex ratio of the offspring. These reproduction assays were conducted with 25 pairs of insects per replicate, with three replicates, for a total of 75 pairs per bean seed.

### 2.5. Population Performance of C. chinensis

Ten pairs of *C. chinensis* adults (1–2 days after emergence) were introduced to a jar (2.5 L) containing one of the stored beans (*V. radiata*, *P. sativum*, *V. unguiculata*, *V. faba*, and *G. max*) at 200.0 g, using different jars for each bean seed. The jar openings were covered with a 60-mesh gauze to prevent insects from escaping. The number of *C. chinensis* adults was recorded in the F_1_, F_2_, and F_3_ generations. Generations were based on the eclosion (i.e., the emergence of adult progeny), with one generation in these trials lasting approximately 30 days. For each generation on each sampling date (30, 60, and 90 days), *C. chinensis* adults were counted to evaluate the increase in the population at each generation. Treatments were replicated three times for each bean species.

### 2.6. Enzyme Extract Preparation

Enzyme extracts were prepared from *C. chinensis* fed on each of these five bean seeds. Following our previously described protocols, protein extraction and a total protein quantitative assay were performed (Nanjing Jiancheng Bioengineering Institute, Nanjing, China) [14,16]. In brief, randomly selected *C. chinensis* adults or larvae (0.1 g per cultivar) were accurately weighed and placed in centrifuge tubes, followed by the addition of 0.9% normal saline at a 1:9 (*w*/*v*) ratio. The mixture was ground into a homogenate in an ice-water bath, then centrifuged at 2197× *g* and 4 °C for 10 min. The supernatant was collected into 1.5 mL tubes and utilized for the determination of enzyme activities in subsequent experiments. Protein concentrations were determined according to the Bradford method using bovine serum albumin as the standard [17].

### 2.7. Digestive Enzyme Activity Assay

Activities of digestive enzymes were tested following the methods detailed in our previous study using activity assay kits for *α*-amylase (*α*-AMS), pepsin (PEP), lipase (LPS), and cellulase (CL), according to the manufacturer’s instructions [14,16]. The activity levels of LPS, *α*-AMS, CL, and PEP were calculated by measuring the increase in optical density at 420 nm, 660 nm, 550 nm and 270 nm, respectively. The optical density was recorded using a microplate reader. Three repetitions were conducted for each digestive enzyme, and larvae and adults of *C. chinensis* were tested.

### 2.8. Statistical Analysis

One-way analyses of variance (ANOVA) followed by Tukey’s honestly significant difference (HSD) tests were used for comparisons of development, survival, oviposition parameters, and digestive enzyme activities in *C. chinensis* among different stored beans (*p* < 0.05). All data were checked for normality and homoscedasticity before analyses. Data were analyzed using SPSS software (version 22.0; SPSS, Chicago, IL, USA).

## 3. Results

### 3.1. Development

The developmental periods of *C. chinensis* eggs (*F*_4,10_ = 29.28, *p* < 0.01) and larvae and pupae (*F*_4,10_ = 34.75, *p* < 0.01) differed significantly among the five bean seeds (Table 1). There were also significant differences in the duration of development (egg to adult) (*F*_4,10_ = 75.99, *p* < 0.01) in *C. chinensis*, with estimates of 26.97 days on *V. radiata*, 27.82 days on *P. sativum*, 28.43 days on *V. unguiculata*, 29.20 days on *V. faba*, and 30.92 days on *G. max*.

### 3.2. Emergence Rate

**Figure 1:**
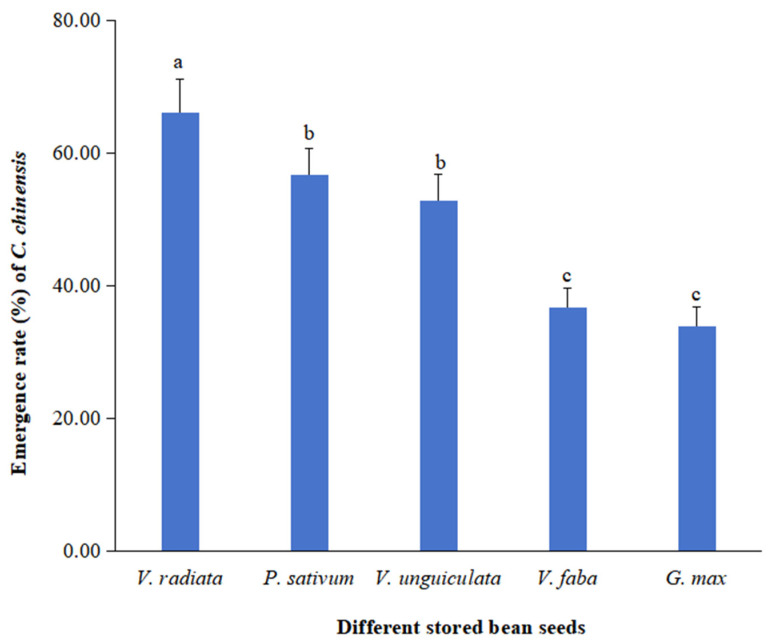
Emergence rate (%) of *C. chinensis* raised on different bean seeds. Data are presented as means ± SE. Different letters above bars indicate significant differences among values (Tukey’s test, *p* < 0.05).

The emergence rate of *C. chinensis* (egg to adult) (*F*_4,10_ = 201.55, *p* < 0.01) differed significantly among the five selected stored products (Figure 1). *C. chinensis* adults showed the highest emergence rate (66.11%) on *V. radiata*, followed by 56.67% on *P. sativum* and 52.78% on *V. unguiculata*, 36.67% on *V. faba,* and 33.89% on *G. max*.

### 3.3. Oviposition

The type of bean seed had significant effects on the longevity of adult females (*F*_4,10_ = 16.45, *p* < 0.01) and males (*F*_4,10_ = 23.80, *p* < 0.01) of *C. chinensis* (Table 2). Fecundity also differed significantly among bean types (*F*_4,10_ = 306.96, *p* < 0.01). The stored beans were ranked, from highest to lowest with respect to the fecundity of *C. chinensis*, as follows: *V. radiata* (63.36), *P. sativum* (54.27), *V. unguiculata* (51.30), *V. faba* (42.47), and *G. max* (37.29). There were significant differences in the oviposition periods of *C. chinensis* among these five bean seeds (*F*_4,10_ = 75.20, *p* < 0.01), with the longest period on *V. radiata* (10.98 days) and the lowest on *G. max* (7.18 days), which was not significantly different from that on *V. faba* (7.54 days). In addition, the female offspring sex ratio for *C. chinensis* was highest on *V. radiata* (0.54), which was not significantly different from those on *P. sativum* (0.52) and *V. unguiculata* (0.51). The sex ratio was lowest on *G. max* (0.43), which was not significantly different from that on *V. faba* (0.45) (*F*_4,10_ = 18.47, *p* < 0.01).

### 3.4. Population Performance

**Figure 2:**
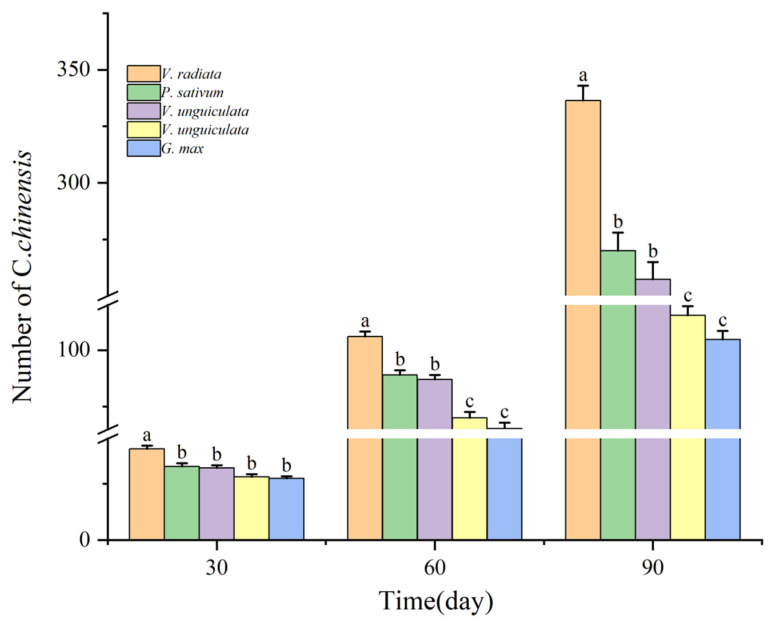
Population size of *C. chinensis* raised on different bean seeds. Data are presented as means ± SE. Different letters above bars indicate significant differences among values (Tukey’s test, *p* < 0.05).

After 30 days, the number of *C. chinensis* adults was significantly higher on *V. radiata* (40.33) than on other bean species, and there were no significant differences in the number of *C. chinensis* adults among the other four stored beans (*F*_4,10_ = 17.55, *p* < 0.01) (Figure 2). Generally, the population sizes on each bean seed were 2.02, 1.63, 1.60, 1.40, and 1.37 times greater than the initial number of *C. chinensis* on *V. radiata*, *P. sativum*, *V. unguiculata*, *V. faba*, and *G. max*, respectively.

After 60 days, there were significant differences in the number of *C. chinensis* adults among the five stored bean products (*F*_4,10_ = 47.75, *p* < 0.01), with the highest number of 106.00 on *V. radiata*, followed by *P. sativum* (89.00), *V. unguiculata* (87.00), *V. faba* (70.00), and *G. max* (65.33). The population sizes on each stored bean species were 5.30, 4.45, 4.35, 3.50, and 3.27 times greater than the initial number of *C. chinensis*, respectively.

After 90 days, there were significant differences in the number of *C. chinensis* adults among these stored beans (*F*_4,10_ = 264.45, *p* < 0.01), with the highest number of 336.33 on *V. radiata*, followed by 270.00 on *P. sativum,* 257.33 on *V. unguiculata*, 115.55 on *V. faba,* and 104.67 on *G. max.* The population sizes on each stored bean were 16.82, 13.50, 12.87, 5.77, and 5.23 times greater than the initial number of *C. chinensis*, respectively.

### 3.5. Digestive Enzyme Activity

**Figure 3:**
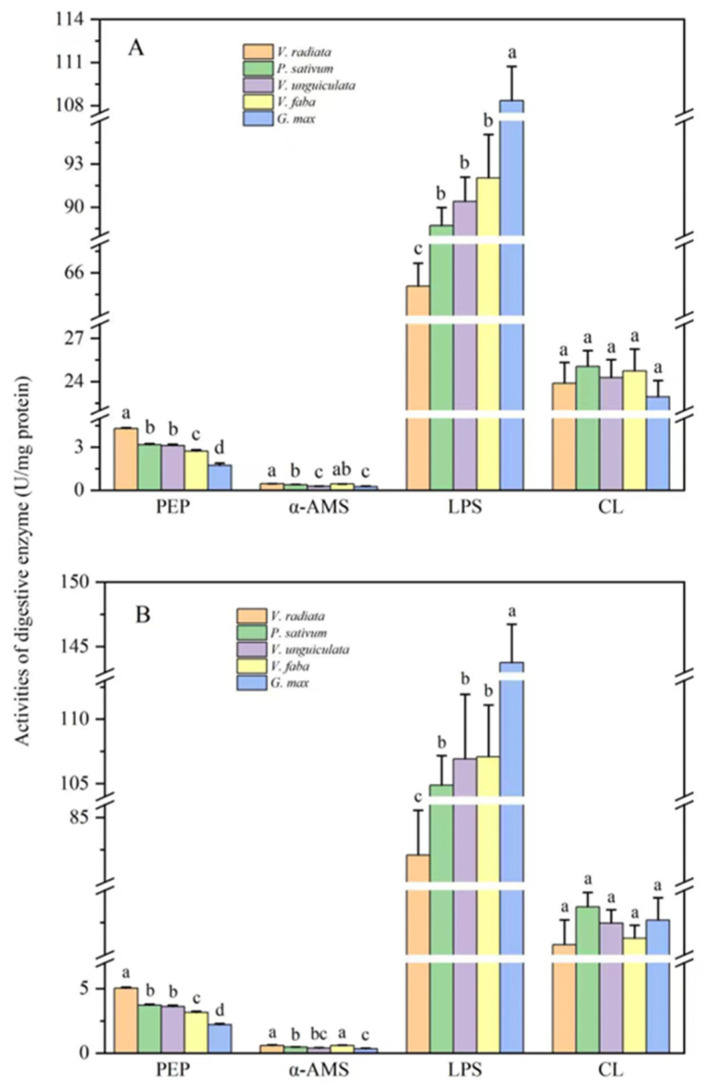
Digestive enzyme activities in *C. chinensis* raised on different bean seeds. Data are presented as means ± SE. Different letters above bars indicate significant differences among values (Tukey’s test, *p* < 0.05). (**A**) adults; (**B**) larvae.

PEP activity in *C. chinensis* adults differed significantly among bean seeds (*F*_4,10_ = 258.67, *p* < 0.01). PEP activity was highest on *V. radiata* at 4.30 U/mg∙prot, which was 1.36, 1.38, 1.58, and 2.47 times greater than those on *P. sativum*, *V. unguiculata*, *V. faba*, and *G. max*, respectively (Figure 3A). *α*-AMS activity of *C. chinensis* adults was highest on *V. radiata* at 0.45 U/mg∙protein, which was not significantly different from that on *V. faba* (0.43 U/mg∙protein). *α*-AMS activity was lowest on *G. max* at 0.26 U/mg∙protein, which was not significantly different from that on *V. unguiculata* (0.27 U/mg∙protein) (*F*_4,10_ = 49.47, *p* < 0.01). Significant differences in LPS activity were also observed among stored beans, with the highest values on *G. max* (108.35 U/mg∙protein) and the lowest values on *V. radiata* (65.07 U/mg∙protein) (*F*_4,10_ = 164.91, *p* < 0.01). There were no significant differences in LPS activity in *C. chinensis* among the other three stored beans. In addition, no significant differences in CL activity were observed among adult *C. chinensis* on different stored beans (*F*_4,10_ = 1.23, *p* = 0.36).

In *C. chinensis* larvae, there were significant differences in the activities of PEP (*F*_4,10_ = 378.39, *p* < 0.01), *α*-AMS (*F*_4,10_ = 41.52, *p* < 0.01), and LPS (*F*_4,10_ = 108.72, *p* < 0.01), but not in CL (*F*_4,10_ = 2.05, *p* = 0.163), among bean seeds (Figure 3B). In addition, activities of PEP, *α*-AMS, LPS, and CL were significantly higher in larvae than in adults on each of these five stored beans.

## 4. Discussion

Previous studies have clearly demonstrated that food source can influence the development, survival, and reproductive characteristics of *C. chinensis* [4,18,19,20]. In the present study, developmental period, emergence rate, fecundity and population growth differed significantly among the five bean seeds, indicating that *C. chinensis* poses a higher risk to some stored product commodities than the others. This finding should be carefully taken into account, given that certain members of the stored-product Bruchidae are strongly associated with specific legume species, whereas other species, although still oligophagous, can infest a range of legume hosts [21,22]. In this context, categorizing bruchids according to infestation risk in a single commodity may be misleading, as some of these species may have a wider range of food preferences. For instance, the bean weevil, *Acanthoscelides obtectus* (F). (Coleoptera: Bruchidae) is commonly associated with common beans, but has been reported from several other legumes [23,24,25]. Hence, the characterization of this species as a “bean weevil” may be falsely perceived as an indication that *A. obtectus* cannot infest other commodities. Similarly, our results indicate that *G. max* is the least suitable, but *C. chinensis* can still develop on all five legume species, indicating a relatively broad capacity for host utilization.

In the present study, at 25 °C, the development period of *C. chinensis* from egg to adult ranged from 26.97 to 30.92 days on the five selected bean seeds. A similar estimate of 27.01 to 38.2 days was obtained in different *V. radiata* and *V. unguiculata* cultivars under 28 °C [4]. The total developmental period (egg to adult) of *C. chinensis* ranged from 21.8 to 72.7 days under the temperature range of 20~32 °C when fed on *V. radiata* or *V. angularis* [26,27]. There is also evidence that humidity affects the development of *C. chinensis* [20], which is a parameter of major importance in the case of stored product insects [12]. Therefore, additional environmental factors particularly temperature and humidity should be examined to improve prediction of *C. chinensis* development under realistic storage conditions [4,27,28].

Fecundity of *C. chinensis* differed significantly among the five stored beans and was highest on *V. radiata* (63.36 per female) and lowest on *G. max* (37.29 per female), in agreement with the results of Ma et al. [29] and Hao et al. [30]. In other studies, Ge et al. [31] and Maharjan et al. [28] have reported that *C. chinensis* lays more eggs in *V. unguiculata*, *C. arietinum*, and *V. radiata* than on other legumes. In addition, the emergence rate and offspring sex ratio of *C. chinensis* were also higher on these stored products, indicating that *V. radiata*, *V. unguiculata*, and *C. arietinum* are suitable hosts for population growth in *C. chinensis*. In these previous studies, *C. chinensis* had a significantly higher net reproductive rate (*R*_0_) or intrinsic population increase rate (*r*) on *V. radiata*, *V. unguiculata* and *C. arietinum* than on other legumes [29,31]. In this study, *C. chinensis* showed significantly larger population sizes on *V. radiata* than on the other four stored beans after 30, 60, and 90 d. However, the data reported here correspond to the varieties and genotypes tested in our protocols, and thus, broad generalizations should be avoided, even though clear trends in host suitability were observed.

Host quality influences feeding, development, survival, and oviposition of *C. chinensis* [4,18,27,31,32]. The protein content is a crucial factor in *C. chinensis* survival and development because this pest spends the whole immature stage inside the host seed [6,33]. Furthermore, other studies have shown that the degree of damage caused by *C. chinensis* is positively correlated with the protein and starch contents of stored products and is negatively correlated with the flavonoids, phenol, or tannin content of host diets [33,34,35]. In addition, a higher protein content is beneficial for population growth in *C. chinensis*, with significant positive correlations between the protein content of the host and the growth index of *C. chinensis* and negative correlations between these parameters and total phenolic, anthocyanin, and flavonoid contents [36,37]. These nutrients and secondary metabolites contribute to nutrition, and various physiological and biochemical reactions in insects after feeding on these hosts directly affect growth and development [32,35,38]. Future studies should evaluate nutritional composition and secondary metabolites in the same seed lots used for insect bioassays to better clarify their contribution to host suitability and insect performance.

Insects can adapt to different host diets through changes in metabolic processes [15,39,40]. Stored-product bruchids are useful models for studying these adaptations because infestation levels often vary strongly among host seeds [25,41,42]. Digestive enzymes play a crucial role in food digestion, nutrient acquisition, and host utilization [43,44] and may therefore help explain differences in host suitability. In the present study PEP and *α*-AMS activity levels in *C. chinensis* were highest on *V. radiata* and lowest on *G. max*, consistent with the host suitability ranking observed in the development, fecundity, and population performance assays. This could be attributed to the high nutrient contents (protein and starch) and low secondary metabolite contents (phenols, flavonoids, and anthocyanins) of *V. radiata* [45], providing better nutritional value through digestion and metabolism via PEP and *α*-AMS and thereby promoting pest population growth. Although there were no significant differences in CL activity in *C. chinensis* among bean seeds, LPS activity was highest on *G. max* and lowest on *V. radiata*. Overall, these results support the view that variation in digestive enzyme activity is one physiological mechanism underlying host adaptation in *C. chinensis*.

Physical characteristics of seeds can also influence suitability for insect egg laying. Seed size, moisture content, texture, color, and hardness of seeds are important factors affecting *C. chinensis* population growth or host resistance [32,33,36,46]. The high levels of secondary metabolites (phenols, anthocyanins, flavonoids, etc.) make some bean seeds unsuitable hosts for *C. chinensis* [33,34,35,36,37]. In the present study, the selected legumes differed in moisture content, which may have contributed to the observed differences in development, fecundity, and population performance. Further analyses of physical and biochemical properties of the five bean seeds, together with the physiological responses of insects, will aid in comprehensive assessments of the *C. chinensis* population status and host suitability. Physical or biochemical characteristics of bean seeds associated with resistance to *C. chinensis* can be exploited for pest control or IPM programs and could provide useful information for the breeding of resistant cultivars against *C. chinensis* [32,35,36,46].

## Figures and Tables

**Table 1:** Duration (days) of *C. chinensis* development on different bean seeds.

Bean Seeds	Egg	Larval and Pupal	Egg to Adult
*V. radiata*	5.83 ± 0.08 d	21.27 ± 0.32 d	26.97 ± 0.20 d
*P. sativum*	5.90 ± 0.05 cd	22.13 ± 0.27 cd	27.82 ± 0.15 c
*V. unguiculata*	6.17 ± 0.11 bc	22.39 ± 0.30 bc	28.43 ± 0.43 bc
*V. faba*	6.26 ± 0.07 b	23.08 ± 0.39 b	29.20 ± 0.41 b
*G. max*	6.80 ± 0.13 a	24.34 ± 0.40 a	30.92 ± 0.20 a

Data are presented as the mean ± SE. Different lowercase letters in the same column indicate significant differences (one-way ANOVA followed by Tukey’s HSD test, *p* < 0.05).

**Table 2:** Fecundity, offspring sex ratio, and longevity of *C. chinensis* on different bean seeds.

Bean Seeds	Oviposition Days (Day)	Fecundity (Eggs/Female)	Sex Ratio (Females/Total Offspring)	Longevity of Adults (Day)	
				Females	Males
*V. radiata*	10.98 ± 0.35 a	63.36 ± 0.97 a	0.54 ± 0.01 a	15.34 ± 0.36 a	14.01 ± 0.25 a
*P. sativum*	9.27 ± 0.28 b	54.27 ± 0.86 b	0.52 ± 0.01 a	13.45 ± 0.66 b	13.37 ± 0.39 ab
*V. unguiculata*	8.18 ± 0.16 c	51.30 ± 0.69 c	0.51 ± 0.02 a	13.13 ± 0.46 b	12.80 ± 0.26 bc
*V. faba*	7.54 ± 0.37 cd	42.47 ± 1.16 d	0.45 ± 0.01 b	12.16 ± 0.11 bc	12.09 ± 0.30 cd
*G. max*	7.18 ± 0.37 d	37.29 ± 1.26 e	0.43 ± 0.01 b	11.13 ± 0.20 c	11.61 ± 0.26 d

Data are presented as the mean ± SE. Different lowercase letters in the same column indicate significant differences (one-way ANOVA followed by Tukey’s HSD test, *p* < 0.05).

## Data Availability

The original contributions presented in this study are included in the article. Further inquiries can be directed to the corresponding authors.
